# Supplementary material for: The Utility of NGS Analysis in Homologous Recombination Deficiency Tracking
Source: Diagnostics (Basel). 2023 Sep 15;13(18):2962. doi: 10.3390/diagnostics13182962 (PMC10529941; doi:10.3390/diagnostics13182962)
Supplement: Supplementary file 1 [file diagnostics-13-02962-s001.zip › Supplementary Table S1.pdf]

Supplementary Table S1. List of Homologous Recombination genes

ARID1A	CHEK1	FANCL	RAD51C
ATM	CHEK2	FANCM	RAD51D
ATRX	FANCA	MRE11	RAD52
BAP1	FANCC	NBN	RAD54L
BARD1	FANCD2	PALB2	XRCC2
BLM	FANCE	PPP2R2A	XRCC3
BRCA1	FANCF	RAD50	
BRCA2	FANCG	RAD51	
BRIP1	FANCI	RAD51B	
